# How individual participant data meta-analyses have influenced trial design, conduct, and analysis

**DOI:** 10.1016/j.jclinepi.2015.05.024

**Published:** 2015-11

**Authors:** Jayne F. Tierney, Jean-Pierre Pignon, Francois Gueffyier, Mike Clarke, Lisa Askie, Claire L. Vale, Sarah Burdett, Cochrane IPD Meta-analysis Methods Group, P. Alderson, L. Askie, D. Bennett, S. Burdett, M. Clarke, S. Dias, J. Emberson, F. Gueyffier, A. Iorio, M. Macleod, B.W. Mol, C. Moons, M. Parmar, R. Perera, R. Phillips, J.P. Pignon, J. Rees, H. Reitsma, R. Riley, M. Rovers, L. Rydzewska, C. Schmid, S. Shepperd, S. Stenning, L. Stewart, J. Tierney, C. Tudur Smith, C. Vale, J. Welge, I. White, W. Whiteley

**Affiliations:** a MRC Clinical Trials Unit at UCL, Aviation House, 125 Kingsway, London WC2B 6NH, UK; b LNCC plateforme de méta-analyse en oncologie, Service de Biostatistique et d’Epidemiologie, Gustave-Roussy, Villejuif, France; c Université Claude Bernard Lyon 1/Université de Lyon, 69365 Lyon Cedex 07, Lyon, France; d Service de Pharmacologie Clinique, Hospices Civils de Lyon, Bron cedex, France; e All-Ireland Hub for Trials Methodology Research, Queen's University Belfast, University Road, Belfast BT7 1NN, Northern Ireland, UK; f NHMRC Clinical Trials Centre, ABN 15 211 513 464, Locked Bag 77, Camperdown, NSW 1450 Australia

**Keywords:** Individual participant data (IPD), Systematic review, Meta-analysis, Trial conduct, Trial design, Trial analysis

## Abstract

**Objectives:**

To demonstrate how individual participant data (IPD) meta-analyses have impacted directly on the design and conduct of trials and highlight other advantages IPD might offer.

**Study Design and Setting:**

Potential examples of the impact of IPD meta-analyses on trials were identified at an international workshop, attended by individuals with experience in the conduct of IPD meta-analyses and knowledge of trials in their respective clinical areas. Experts in the field who did not attend were asked to provide any further examples. We then examined relevant trial protocols, publications, and Web sites to verify the impacts of the IPD meta-analyses. A subgroup of workshop attendees sought further examples and identified other aspects of trial design and conduct that may inform IPD meta-analyses.

**Results:**

We identified 52 examples of IPD meta-analyses thought to have had a direct impact on the design or conduct of trials. After screening relevant trial protocols and publications, we identified 28 instances where IPD meta-analyses had clearly impacted on trials. They have influenced the selection of comparators and participants, sample size calculations, analysis and interpretation of subsequent trials, and the conduct and analysis of ongoing trials, sometimes in ways that would not possible with systematic reviews of aggregate data. We identified additional potential ways that IPD meta-analyses could be used to influence trials.

**Conclusions:**

IPD meta-analysis could be better used to inform the design, conduct, analysis, and interpretation of trials.

**What is new?**

• Systematic reviews and meta-analyses based on aggregate data can inform subsequent clinical trials, but empirical evidence of this happening is limited.
• Systematic reviews and meta-analyses based on IPD are international collaborative projects that often provide more detailed and reliable results and so have greater potential to inform trials.
• To our knowledge, this is the first attempt to explore the impact of IPD meta-analyses on ongoing and subsequent trials.
• We identified examples of IPD meta-analyses having a direct impact on trial design, conduct, analysis, and interpretation, sometimes in ways that would not be possible with aggregate data.
• IPD meta-analysis could be better used to inform additional aspects of design and conduct.

## 1 Introduction

Systematic reviews are recognized as the optimal way to resolve or confirm uncertainty about the effects of interventions, both informing clinical practice and providing the scientific and ethical justification for the design of new trials [1]. However, empirical evidence suggests that they are still used infrequently to explain the rationale for [2], [3], [4] or directly influence the design of trials [3], [4]. Systematic reviews can also be used to take account of external evidence that accumulates during the conduct of a trial, thereby ensuring that participant recruitment and any protocol amendments are informed by the accumulating external evidence; however, it is not clear how often this actually happens. When a trial is completed, systematic reviews can also help place the results in the context of the results of other related trials, but this is far from standard practice [3], [5], [6].

Most commonly, systematic reviews are based on aggregate data extracted from publications or obtained from trial investigators, which can limit the availability and quality of such data. Furthermore, the range of analyses possible with aggregate data is limited, and they may lack power. Instead, systematic reviews and meta-analyses that involve individual participant data (usually called IPD meta-analyses) tend to be larger scale, international projects in which researchers collaborate to collect and analyze the original data from all the studies relevant to the review question [7], [8], [9]. A well-conducted IPD meta-analysis [10] can bring about substantial improvements to the quantity and quality of the data, for example, by including more eligible trials and participants, and to the analysis, by allowing the investigation of whether treatment effects vary by participant characteristics [8], [9]. Thus, they often provide more detailed and reliable results and a greater depth of understanding than is possible from aggregate data. This has led to them being coined the “gold standard” of systematic review [11]. Collated IPD also represents a unique resource for secondary hypothesis testing and exploratory analyses, which can provide further clinical insight. Thus, IPD meta-analyses have the potential to inform the design, conduct, analysis, and interpretation of subsequent trials in ways that are IPD specific, as well as in ways that would also be possible aggregate data. This article aims to provide verified examples of both. Given that IPD meta-analyses can take longer and be more resource intensive than standard systematic reviews based on aggregate data, we also want to highlight how IPD meta-analyses might be better used to inform ongoing or new trials.

## 2 Methods

Funders of randomized trials often require that relevant systematic reviews are cited and used in the trial funding application, and this direct linkage has allowed researchers to use cohorts of such applications to assess the impact of aggregate data systematic reviews on trial design and conduct [4]. It would not be possible to define a similar cohort of trials and trace the impact of IPD meta-analyses on these, as IPD meta-analyses are not a requirement for trial funding and remain relatively few in number. Therefore, rather than comprehensively identifying the effects of IPD meta-analyses on trials, we aim to provide a range of illustrative examples. Initially, we sought examples at an international workshop of 31 members of the Cochrane IPD Meta-analysis Methods Group, which took place in London in September 2012. Not only did the attendees have considerable experience in the conduct of IPD meta-analyses, but also actively collaborate with trialists as part of the IPD approach and have valuable knowledge of trials in their respective clinical areas. In March 2015, we also surveyed the entire membership of the Methods Group to solicit further examples. Two authors (J.F.T. and S.B.) screened relevant trial protocols, publications, and Web sites to verify those IPD meta-analyses that were used to support trial design and conduct (Box 1, Box 2, Box 3 and Table 1). A subgroup of workshop attendees (the authors) sought further examples of impact from their own experience and identified aspects of trial design and conduct that might better informed by IPD meta-analyses.

## 3 Results

We identified 52 IPD meta-analyses thought to have had direct impacts on the design and conduct of trials. On further investigation, we could find no evidence of a link between 24 IPD meta-analyses and trials but that is not to say that such a link does not exist. For five of these examples, we suspect that it was too soon after the publication of the IPD meta-analysis for a trial to have credited it. Therefore, we found 28 IPD meta-analyses giving 29 instances where the trial protocol, publication, or Web site explicitly described how an IPD meta-analysis had influenced the trial. Sometimes more than one IPD meta-analysis contributed to an impact or more than one impact was derived from a single IPD meta-analysis. In 19 cases, these impacts would only be possible with IPD, either because the results or other aspects were IPD specific (Box 1, Box 2, Box 3, Table 1). For the remaining 10, the impacts we identified might also have been possible with an equivalent aggregate data meta-analyses (Box 1, Table 1). This cohort of examples has also helped highlight the ways in which IPD meta-analyses might be underused in trial design and conduct.

### 3.1 Impact on trial design

#### 3.1.1 Choosing comparators

Systematic reviews and meta-analyses based on aggregate data have the potential to influence the choice of comparators in a subsequent trial, and there is already some evidence of this [4]. We found too that IPD meta-analyses showing a clear, robust, and clinically relevant effect of an intervention have been used to justify use of the intervention as a control group comparator in subsequent randomized trials (breast cancer 1, Table 1). Even when results of an IPD meta-analysis were less definitive but suggested that an intervention might be beneficial, we saw that this can maintain or generate interest in particular therapeutic comparisons, giving the impetus both for ongoing trials to continue and for confirmatory or trials to be conducted (NSCLC 1, Table 1). Instead, an IPD meta-analysis that fails to show any benefit or shows that a therapy is harmful could provide the rationale for discontinuing its further investigation and for the current standard therapy to remain as a control against which emerging therapies should be compared, but we did not find evidence of the overall results of a meta-analysis being used in this way. Although we suspect that this is because it is easier to justify maintaining the status quo, without explicit reference to a systematic review, ideally the current evidence should be cited.

#### 3.1.2 Promoting consensus and collaboration

The collaborative group needed to conduct an IPD meta-analysis often includes those investigators who have supplied their trial data, and we have found that this can facilitate consensus being reached on the design of subsequent trials (Box 1). It is common practice to bring together such a group of individuals, from different clinical specialties and disciplines to discuss the preliminary results of an IPD meta-analysis and their implications. As this is often in advance of formal presentation or publication of the results, it can also help speed up the design and launch of a new trial (Box 1) or even refine the design of one still in development (soft tissue sarcoma 1, Table 1). Moreover, a group of trialists are well placed to judge how a new trial might be achieved and by whom. In one example, where large-scale international collaboration was needed to achieve a trial of sufficient size (to detect a small predicted treatment effect), it was drawn from the meta-analyses collaborative groups (Box 1); an advantage likely restricted to the IPD approach.

#### 3.1.3 Determining sample size

A new trial can draw on estimates of control group or baseline risk provided by a meta-analysis to give a good indication of the approximate effect size to target in trials of new or confirmatory comparisons. Examples of these sorts of impact are evident for both IPD (NSCLC 1, cervix I, Table 1) and aggregate data reviews [4]. It would be possible to interrogate the IPD further to provide estimates of baseline risk for particular types of participant, such as those who might be expected to benefit most from a new therapy, thereby helping to refine or improve the reliability of sample size calculations. IPD could also be used to inform predictions of trial duration and follow-up and hence, resourcing, we did not find examples of IPD meta-analyses being exploited to this extent.

#### 3.1.4 Defining the population

Pooling IPD from multiple trials provides considerably more power than any individual trial to investigate how participant or disease characteristics interact with treatment effects, and uniquely, the IPD also allow the relative influence of multiple trial and participant characteristics to be considered simultaneously, which can help deal with confounding [53]. Not surprisingly then, IPD meta-analyses showing interactions between treatment effects and particular participant or disease characteristics have been used to justify focusing on particular subgroups or characteristics in subsequent trials (e.g., hypertension 1 and head and neck 1, Table 1). Although being able to establish with confidence that an effect is not modified by a participant or disease characteristics can reassure trialists that the inclusion criteria should remain broad for the next trial, we did not find evidence of IPD meta-analyses results being used in this way.

IPD collected for a meta-analysis also provide a resource for assessing the prognostic effects of individual characteristics or for defining risk groups based on multiple prognostic factors. In particular, detailed IPD from multiple studies offer the possibility to both generate and validate prognostic models within the same meta-analysis or to validate already established prognostic standards (Box 2), which could be used to inform the stratified randomization procedures in subsequent trials, although we have not found any examples of this. As aggregate data meta-analyses usually cannot be used to determine appropriately prognostic effects or interactions between treatment effects and individual participant characteristics [54], [55], we would not expect them to affect trials in these ways.

#### 3.1.5 Choosing outcomes

There is certainly evidence that aggregate data reviews have helped inform the choice of outcomes in new trials [4]. However, a major advantage of making such decisions based on an IPD meta-analysis is that often it will include data on more outcomes per trial and more complete information on those outcomes, and therefore, results are less likely to be skewed by the biases associated with selective reporting of outcomes [56]. For example, when an IPD meta-analysis establishes that benefits of treatments are consistent across related outcomes, it can provide extra reassurance that a certain intervention should be used in subsequent trials (head and neck 1, NSCLC 1, Table 1). In contrast, the identification of differential effects of an intervention across related outcomes, with IPD, can give a clearer picture of its mode of action and more specifically inform trial design (head and neck 1, Table 1). As underreporting of harms of interventions is particularly prevalent in RCTs [57], perhaps only an IPD meta-analysis including comprehensive data on such outcomes and so properly ascribing the risks of a therapy can properly inform this aspect of the design of subsequent trials. Less obvious perhaps is that the process of seeking data from multiple trials for an IPD meta-analysis can highlight not only those outcomes that were underreported, but also those that that were not collected routinely, so providing evidence on where there is room for improvement [31]. However, we did not find examples of IPD meta-analyses being used to inform these sorts of decisions in new trials.

Although aggregate data can be used to determine whether the effects of an intervention on a proposed surrogate and final outcome are correlated, associations between these outcomes using participant-level data are considered necessary for proper surrogate validation [58]. Thus, data from one or more IPD meta-analyses can be used as a resource to identify reliable surrogate outcomes, as well as those that are clearly unsuitable. In principle, this might identify shorter-term surrogates with the potential to speed up the evaluation of therapies in new trials. Alternatively, finding a reliable surrogate outcome that is more practical to measure could facilitate outcome data collection and so improve data quality in subsequent trials. Although IPD meta-analyses have been used to validate surrogate outcomes [59], [60], in our cohort, we did not find evidence of trials adopting such surrogate outcomes on the basis of these analyses. One possible explanation is that the treatments investigated in the new trials are different to those evaluated in prior meta-analysis, such that further validation would be necessary.

#### 3.1.6 Defining and collecting outcome data

If outcomes have been defined and analyzed very differently in individual trials, it may be necessary to make assumptions about their comparability or use standardized effect measures in aggregate data meta-analysis, whereas IPD offer the opportunity to translate varying outcome definitions into an agreed common scale or to generate standardized definitions from other data items [61]. Thus, if an IPD meta-analysis can demonstrate effects of a therapy based on such a common or standardized outcome definition, it provides trialists with both the rationale and motivation for adopting a standard definition in future trials. Moreover, the process of collecting IPD can reveal outcomes or outcome definitions that generate better or poorer quality data, which could also help optimize how data are defined and collected in the next generation of trials. To our knowledge, however, these advantages of IPD have not been explicitly described in the design of RCTs.

For time-to-event outcomes, a major advantage of collecting IPD is the ability to obtain additional follow-up from investigators, sometimes substantially beyond the aggregate data results reported in trial publications. Although the primary aim is to provide more events and greater power for the analysis, importantly, this practice can help reveal the pattern of events over time. In particular, any benefits or harms of interventions that take a long time to accrue, such as late side effects of treatment or late recurrence of disease, can become apparent. We did find an example, where the IPD meta-analysis highlighted the need to collect long-term outcome data in subsequent trials, as well as informing the appropriate duration of data collection (breast cancer 1 Table 1).

### 3.2 Impacts on trial conduct

#### 3.2.1 Informing the conduct of ongoing trials

For trials that are ongoing, a prospective approach to IPD meta-analysis can offer greater collective power to produce definitive results when, for example, it has not been possible to adequately power individual trials for all the outcomes of interest and/or there are practical or other barriers to adhering to a single trial protocol. We also found a prospective design being adopted, when sufficiently powered trials recruited less well than expected (Box 3, colon cancer 1, Table 1), or the prognosis of the participants' was more favorable than predicted at the design stage (Box 3), such that events accrued more slowly than anticipated. In one example, these issues would have affected the timely completion of the individual trials, reduced their power to detect effects of therapies reliably, and jeopardized their continuation (Box 3). A prospective decision to pool the trials has been used to encourage continued recruitment into the individual trials and justify their ongoing follow-up and funding (Box 3). A major advantage of prospective meta-analysis is that hypotheses, inclusion criteria, and analyses are defined before, and without influence from, the results of individual trials, which is a potential source of bias for retrospectively designed IPD meta-analyses [62]. Although individual trials often run according to their own protocols and timetables, there is also the potential for the proposed meta-analysis to directly influence the day-to-day running of the individual studies. For example, we found that investigators may align trial materials and procedures (stroke 1, preterm neonates 1, Table 1). A clear challenge of this approach, however, is being able to respond to the accumulating results of the individual trials, and managing their impact on the remaining ongoing trials, the meta-analysis collaboration, and subsequent meta-analysis results.

#### 3.2.2 Stopping ongoing trials

In a similar way to a standard systematic review, an IPD meta-analysis that shows that an intervention is inferior to standard treatment, or harmful, should discourage further use of that intervention both in practice and in future trials. Of course, a comprehensive IPD meta-analysis may show this more definitively and reliably, or, importantly, for a certain subgroup of participants (NSCLC 1, Table 1).

### 3.3 Impacts on trial analysis and interpretation

#### 3.3.1 Informing the analyses

Prognostic factors or risk groups identified via IPD have been used to adjust or stratify trial analyses (Box 2), as well as being used to define the population to be studied in a new trial. In addition, where an IPD meta-analysis has identified participants or disease characteristics that seem to modify treatment effects, this had led to further exploration and testing in subsequent trials, for example, by stratifying the analysis by these characteristics (Box 2).

#### 3.3.2 Interpreting and reporting results

Trial results can be readily placed in the context of the results of an existing meta-analysis using standard two-stage techniques, and is possible both with aggregate data [3], [5] and IPD. The latter does not necessarily require access to the full IPD, but rather the reported results of the IPD meta-analysis. In fact, for the examples we identified, the trial results were combined both with the results of an existing IPD meta-analysis and results of other trials published subsequently, to provide the totality of evidence about intervention effects. Anecdotal evidence also suggests that participation in an IPD meta-analysis encourages trialists to publish previously unreported trials or to publish updated analyses.

### 3.4 Potential negative impacts on trials

As IPD meta-analyses can take years from inception to final publication, awaiting the final results can delay the start of a new trial by a similar time frame. Moreover, if the results suggest benefit or harm of a particular intervention, even if not definitively, this can jeopardize recruitment to ongoing trials investigating that intervention.

## 4 Discussion

Through consultation with experts in the field, we have identified a cohort of 29 examples of IPD meta-analyses impacting directly on trials. They have influenced the selection of comparators and sample size calculations of subsequent trials, and also been used to place trial results in the context of the other evidence in a similar fashion to aggregate data reviews [4]. In addition, IPD meta-analyses have played a role in the selection of participants, and in the conduct, analysis, and interpretation of trials, particularly in response to subgroup or prognostic factor analyses, neither of which are not possible with aggregate data. This study also highlights that IPD meta-analyses generate information, for example, on the natural history of disease or on the definition of outcomes that could be used to inform trials, but we have yet to find evidence of this.

To our knowledge, this is the first attempt to explore how IPD meta-analyses can impact on subsequent trials. By considering relevant trial protocols, publications, and Web sites, we have ensured that the examples presented here are evidence based. That does not mean that the other IPD meta-analyses we examined have not had an impact on trials but rather that no evidence of this could be found. Having targeted a selected group of individuals with experience in the conduct of IPD meta-analyses, many with knowledge of related trials, means that the study represents only a small subset of potential impacts. Indeed, many of the examples are in the cancer and cardiovascular fields, where there is a long history of using the IPD approach. In other clinical areas, the collection of IPD for meta-analysis has been a more recent phenomenon, and so, it could be some time before any influence on trials becomes apparent. Although our list of examples is illustrative rather than exhaustive, it does emphasize potential missed opportunities to use retrospective or prospective IPD meta-analysis in trial design, conduct, analysis, and interpretation.

Those undertaking IPD meta-analyses might maximize their impact on trials by publishing as speedily as possible, following an open-access model, to ensure widespread availability of results. Making full use of journal online facilities and using reporting guidelines for systematic reviews based on IPD [63] should ensure such reports are sufficiently detailed and help trialists to select those IPD meta-analyses that are well conducted [10]. The outputs and recommendations in IPD meta-analysis reports tend to be focused on clinical practice, so perhaps there is a need to regard IPD meta-analyses as playing an equally important role in informing subsequent clinical research. Structuring recommendations on the direction of new research around the quality of the existing evidence, and the interventions, comparisons and outcomes [63], [64], would highlight gaps and areas for improvement. Ideally, such recommendations would draw on the spectrum of expertize found among trialists in an IPD collaborative group, who will often be key players in the next generation of trials. Furthermore, if these collaborations could also be harnessed to plan and conduct future studies more strategically, it might avoid the duplication of effort and inadequately powered trials that remain a feature of some areas of clinical research. Greater involvement of patients and the public in IPD meta-analyses [65] could provide extra insight on which questions are important. Obviously, if the results of an IPD meta-analysis are not definitive, research recommendations should explicitly encourage recruitment to existing relevant trials, so that their continuation is not jeopardized.

Secondary analyses of the IPD, to address additional clinical questions could be potentially very informative to trialists, as well as making maximum use of the IPD collected. Thus, making these a natural part of the process would be desirable. Certainly, those conducting trials might be able to help direct this kind of research by highlighting, for example, that surrogate outcomes or potential prognostic factors need evaluating. Also, where possible, IPD meta-analysts should be amenable to requests for extra information or analyses that might help trialists refine the design and conduct of further trials.

Many trials do not appear to take prior trials [66] or systematic reviews [4] into account, so we encourage more widespread use of evidence synthesis to inform all stages of trials. Although we identified examples of IPD meta-analyses having a direct impact on trials, sometimes in ways not possible with aggregate data, the richness of the results and the underlying data, and the collaborative advantages seem to be underused. The potential benefits of both retrospective and prospective IPD meta-analyses to clinical research need to be more widely recognized, especially as these projects are likely to benefit from a drive for greater sharing of data held within trials [67]. Thus, wherever available, well-conducted IPD meta-analyses should be used to inform trials.

## Figures and Tables

**Table 1:** Further examples of the impact of IPD meta-analyses on trial design, conduct, analysis, and interpretation

Example	Description of IPD meta-analyses	What was the impact?	Impact IPD specific
*Trial design*			
Breast cancer [1]	• 3 IPD meta-analyses comparing effects of hormonal therapy vs. non or two durations of this treatment for early breast cancer [19]. • 71 Trials and 80,273 patients.	• Informed choice of comparators in the ATLAS trial [20], [21].	• No

Non–small cell lung cancer [1]	• 4 IPD meta-analyses investigating effects of adding chemotherapy to surgery; surgery and radiotherapy; radiotherapy and supportive care [22]. • 52 Trials and 9,387 patients.	• Informed the choice of comparators in new trials in locally advanced disease [23], [24]. • Renewed enthusiasm for chemotherapy led to collaborations on new trials from IPD collaborative group [25], [26], [27], [28]. • Control group survival and absolute survival benefits used as the basis for sample size calculation [27], [28].	• No • Yes • No

Soft tissue sarcoma [1]	• 1 IPD meta-analysis investigating the effects of adding doxorubicin-based chemotherapy after local treatment [29]. • 14 Trials and 1,568 patients.	• Subgroup results helped define population in the RTOG 95-14 trial [30].	• Yes

Cervical cancer [1]	• 1 IPD meta-analysis investigating concomitant chemoradiotherapy vs. the same radiotherapy [31]. • 18 Trials and 4,818 patients.	• IPD meta-analysis and another trial together informed choice of comparators in the OUTBACK trial (ANZGOG 0902/GOG-0274/RTOG 1174). • Control group survival used as the basis for sample size calculation in the OUTBACK trial.	• No • No

Hypertension [1]	• 1 IPD meta-analysis investigating antihypertensive drugs in very elderly patients [32]. • 7 Trials and 1,670 patients.	• Subgroup results helped define the population in the HYVET trial [33], [34].	• Yes

Hypertension [2]	• 1 IPD meta-analysis investigating the effect of antihypertensive treatment in patients having already suffered a stroke [35]. • 9 Trials and 6,752 patients.	• Subgroup results helped define population for the PROGRESS trial [36].	• Yes

Hypertension [3]	• 1 IPD meta-analysis investigating diuretic vs. placebo-based treatment of hypertension for diabetes [37]. • 4 Trials and 18,097 patients.	• Subgroup results helped define population for the ADVANCE trial [38], [39].	• Yes

Head and neck [1]	• 1 IPD meta-analysis comparing conventional radiotherapy vs. altered fractionated radiotherapy for head and neck cancer [40]. • 15 Trials and 6,515 patients.	• Subgroup results helped define population of GORTEC-ELAN-RT (NCT01864850) trial.	• Yes

Small-cell lung cancer [1]	• 1 IPD meta-analysis comparing prophylactic cranial irradiation (PCI) vs. none in patients with small-cell lung cancer [41]. • 7 Trials and 987 patients.	• Informed choice of comparators in PCI 99-01/EORTC 22003-08004/RTOG 0212/IFCT 99-01 trial [42].	• No

Breast cancer [2]	• 1 IPD meta-analysis comparing radiotherapy and other treatments vs. the same other treatment with no radiotherapy [43]. • 40 RCTs and 19,582 patients.	• Informed choice of comparators in SUPREMO trial (ISRCTN 61145589).	• No

Non–small cell lung cancer [2]	• 1 IPD meta-analysis of postoperative radiotherapy vs. none [44]. • 11 RCTs and 2,343 patients.	• Subgroup results helped define population in the Lung ART trial (NCT00410683)	• Yes

Stroke [1]	• Prospective IPD meta-analysis comparing antidepressants vs. none in recovering stroke patients. • 2 Trials and 4,600 patients (approx.).	• Prospective IPD meta-analysis led to common trial design of the two included trials ○ Affinity—www.affinitytrial.org ○ FOCUS—www.focustrial.org.uk	• Yes

Preterm neonates	• Prospective IPD meta-analysis comparing oxygen saturation given to extremely premature babies [45]. • 5 Trials and 5,000 patients (approx).	• Prospective IPD meta-analysis to achieve power for key outcomes.	• Yes

*Trial conduct*			
Colon cancer [1]	• Prospective IPD meta-analysis comparing adjuvant fluorouracil and folinic acid vs. control for Dukes B and C colon cancer [46]. • 3 Trials and 1,493 patients.	• Prospective IPD meta-analysis used to achieve power for key outcome.	• Yes

Stroke [1]	• As above	• Collaborative design of prospective IPD meta-analysis study materials in the two included trials.	• Yes

Non–small cell lung cancer [3]	• 1 IPD meta-analysis of postoperative platinum-based chemotherapy vs. none for NSCLC [47]. • 2 Trials and 494 patients.	• Subgroup results used as rationale for trial stopping (IFCT 0801, TASTE (NCT00775385 [48], [49].	• Yes

Preterm neonates	• As above	• Prospective IPD meta-analysis influenced aspects of the individual trials, for example, data collection and study materials.	• Yes

*Trial analysis and interpretation*			
Soft tissue sarcoma [2]	• IPD meta-analysis investigating the effects of adding doxorubicin-based chemotherapy after local treatment [29]. • 14 Trials and 1,568 patients.	• EORTC 62931 trial report uses results of meta-analysis and subsequent trials to places trial results in context [50].	• No

Stroke [1]	• As above	• Prospective IPD meta-analysis influenced planned analysis of two included trials.	• Yes

Bladder cancer	• IPD meta-analysis of adjuvant chemotherapy vs. none in bladder [51]. • 6 Trials and 491 patients.	• EORTC 30994 trial report [52] uses results of IPD meta-analyses and subsequent results to places trial results in context.	• No

*Abbreviations*: IPD, individual participant data; RCT, randomized controlled trial.

## Box 1 Impact on trial design: choice of comparators, and reaching consensus and collaboration on the next trial

**The individual participant data (IPD) meta-analyses**
• Five meta-analyses comparing the effects of different types of chemotherapy for advanced ovarian cancer [12].
• One related meta-analysis comparing CAP (cyclophosphamide, doxorubicin, and cisplatin) with CP (cyclophosphamide and cisplatin) chemotherapy [13].
• Together included ∼50 trials and ∼10,000 women.
• Provided the first comprehensive synthesis of the effects of chemotherapy for advanced ovarian cancer.

**The pertinent results**
• Platinum-based chemotherapy better than nonplatinum chemotherapy [12].
• Platinum-based combination chemotherapy better than single-agent platinum-based chemotherapy [12].
• No clear difference between the effects of carboplatin and cisplatin when used as single agents or when substituted for one another in combination regimens [12].
• CAP better than CP but dose of cisplatin in CAP regimen suboptimal because of toxicity [13].

**Impact on new trials**
(1) Informed choice of comparators in new trial.
  • International Collaborative Ovarian Neoplasm 2 trial (ICON2) compared CAP with optimal dose carboplatin [14]; the latter being less toxic than cisplatin.
  • In its time, ICON2 was the largest trial to be conducted in ovarian cancer and it showed definitely that carboplatin was an effective but less toxic treatment than CAP for women with advanced ovarian cancer.
  • Carboplatin was the control group comparator in the subsequent ICON3 trial [15].
  • Impact may have been possible using aggregate data.
(2) Collaboration
  • Large-scale collaboration was needed to achieve a trial of sufficient size and was formed from the meta-analyses collaborative groups.
  • A total of 1,526 patients in ICON2 were recruited by 132 centers in nine countries.
  • Collaborations and strategic planning of ovarian cancer trials (ICON 1–8) have continued long after the original meta-analysis.
  • Impact only possible because of the individual participant data approach.

## Box 2 Impact on trial analysis and interpretation: validated prognostic factor used to stratify new trial analysis, which formed part of the final report

**The individual participant data (IPD) meta-analysis**
• Eleven acute stroke trials, 5,843 participants fitting entry criteria [16] from the Virtual International Stroke Archive (VISTA, www.gla.ac.uk).

**Pertinent results**
• Prognostic model developed and validated using IPD.
• Provided the predicted probability of a poor outcome at 6 months [16].

**Impact on new trials**
(1) Prognostic factor analysis informed trial analysis stratification.
  • Predicted probability of a poor outcome used to stratify the analysis of IST-3 trial of thrombolysis vs. control.
  • Larger benefit of thrombolysis was seen with higher probability of a poor outcome [17].
  • Impact only possible because individual participant data were used.

## Box 3 Impact on trial conduct: designed prospective individual participant data (IPD) meta-analysis of ongoing trials to achieve power, encourage continued recruitment, and inform trial analyses

**The IPD meta-analysis**
• Adjuvant vs. salvage radiotherapy after radical prostatectomy for locally advanced prostate cancer.
• Three trials (RADICALS [ISRCTN40814031], RAVES [NCT00860652], GETUG-17 [NCT00667069]) and ∼2,000 patients

**Impact on ongoing trials**
(1) To achieve power for key outcomes
  • Trials are recruiting slowly and the prognosis of participants is better than expected, so a meta-analysis will help achieve sufficient power to detect an effect on survival.
  • Impact only possible because of individual participant data approach.
(2) Informing the conduct
  • Used to justify and encourage continued recruitment, follow-up, and funding of ongoing trials [18].
(3) Informing the analysis
  • Deciding on definition and analysis of intermediate outcomes.
